# Exploring the role of inflammation in major depressive disorder: beyond the monoamine hypothesis

**DOI:** 10.3389/fnbeh.2023.1282242

**Published:** 2024-01-17

**Authors:** Irene Pastis, Melody G. Santos, Akshita Paruchuri

**Affiliations:** ^1^Department of Psychiatry and Behavioral Medicine, East Carolina University, Greenville, NC, United States; ^2^Internal Medicine and Psychiatry Combined Program, Department of Psychiatry and Behavioral Medicine, East Carolina University, Greenville, NC, United States; ^3^East Carolina University Brody School of Medicine, Greenville, NC, United States

**Keywords:** major depressive disorder, inflammation, monoamine hypothesis, immune pathways, cytokines, kynurenine, toll-like receptor

## Abstract

Major depressive disorder affects approximately 8.4% of the United States population. The World Health Organization estimates that 280 million adults worldwide are suffering from depression. They have estimated that by 2030 it will be the second most serious condition. Current treatment relies on the monoamine hypothesis, however, one-third of patients with MDD do not respond to monoamine-based antidepressants. For years, it was hypothesized that the primary pathway of MDD involved serotonin as the main neurotransmitter. The monoamine hypothesis, a widely accepted theory, sought to explain the biological basis of MDD as being caused by the depletion of monoamine neurotransmitters, namely norepinephrine and serotonin. This hypothesis regarding monoamines as the pathophysiological basis of MDD led to the design and widespread use of selective serotonin reuptake inhibitors. However, given that only one-third of patients improve with SSRI it is reasonable to infer that the pathway involved is more complex than once hypothesized and there are more neurotransmitters, receptors, and molecules involved. The monoamine hypothesis does not explain why there is a delay in the onset of effect and action of SSRIs. Several studies have demonstrated that chronic stress is a risk factor for the development of MDD. Thus the monoamine hypothesis alone is not enough to fully account for the pathophysiology of MDD highlighting the need for further research involving the pathways of MDD. In this paper, we review the role of inflammation and cytokines on MDD and discuss other pathways involved in the development and persistence of depressive symptoms.

## Depression epidemiology and diagnostic criteria

Major depressive disorder (MDD) affects approximately 8.4% of the United States (US) population. Roughly 20 million adults in the US have struggled with MDD at least once in their lives [National Institute of Mental Health (NIMH), n.d.]. The World Health Organization estimates that 280 million adults worldwide are suffering from depression (World Health Organization, 2023). According to a recent study published in the Lancet, depressive disorders had a sharp increase in 2020 since coronavirus disease (COVID) had a global impact on so many people (IHME, 2021). MDD is a disabling condition. It has been estimated by the World Health Organization (WHO) that by 2030 it will be the second most serious condition (Mathers and Loncar, 2006). Current treatment relies on the monoamine hypothesis, however, one-third of patients with MDD do not respond to monoamine-based antidepressants (Berlim and Turecki, 2007). For years, it was hypothesized that the primary pathway of MDD involved serotonin as the main neurotransmitter. The monoamine hypothesis, a widely accepted theory, sought to explain the biological basis of MDD as being caused by the depletion of monoamine neurotransmitters, namely norepinephrine and serotonin (Hirschfeld, 2000). This was initially hypothesized based on studies surrounding the neuroscience of lysergic acid diethylamide (LSD) due to the drug’s peripheral blockade of serotonin receptors and reserpine-induced depletion of serotonin in the brain (Hirschfeld, 2000). Additional evidence was provided upon the discovery of iproniazid, a derivative of isoniazid and antimycobacterial, which was found to improve depressive symptoms in tubercular patients. It was later found that iproniazid was a monoamine oxidase inhibitor and thus prevented the degradation of serotonin and norepinephrine. This hypothesis regarding monoamines as the pathophysiological basis of MDD led to the design and widespread use of selective serotonin reuptake inhibitors (SSRIs) (Hirschfeld, 2000). However, given that only one-third of patients improve with SSRI it is reasonable to infer that the pathway involved is more complex than once hypothesized and there are more neurotransmitters, receptors, and molecules involved. The monoamine hypothesis does not explain why there is a delay in the onset of effect and action of SSRIs. Several studies have demonstrated that chronic stress is a risk factor for the development of MDD (Kendler et al., 1998; Hammen, 2005; Hammen et al., 2009; McLaughlin et al., 2010; Franklin et al., 2018). Thus the monoamine hypothesis alone is not enough to fully account for the pathophysiology of MDD highlighting the need for further research involving the pathways of MDD. Patients with MDD have elevated levels of proinflammatory cytokines, chemokines, and acute-phase proteins (Raison et al., 2006). In this paper, we review the role of inflammation and cytokines on MDD and discuss other pathways involved in the development and persistence of depressive symptoms seen in MDD.

The 12-month prevalence of MDD patients in the US actively treated with pharmacotherapy was 8.9 million and out of them 2.8 million had treatment-resistant MDD. Namely, 30.8% of patients had treatment-resistant MDD (Zhdanava et al., 2021). The purpose of this paper is to review the inflammatory hypothesis and to explain why a subgroup of patients with treatment-resistant depression do not respond to medications based on the monoamine hypothesis. We will review the molecules and biochemical pathways responsible for depressive symptoms in patients with MDD and discuss the role of the inflammatory hypothesis. Inflammation can lead to cytokines directly affecting the metabolism of tryptophan and as a result decreasing the production of serotonin. MDD in the context of inflammation could explain why a subgroup of patients with treatment-resistant depression does not respond to medications that were created based on the monoamine hypothesis and why two-thirds of patients might require anti-inflammatory-immunological-based treatments. Inflammation has been observed to play a striking role in suicidality (Andrew and Miller, 2018). Several studies have examined the role of inflammation and specific cytokine levels in patients who have attempted and in some cases completed suicide. In light of these results, we should reconsider our initial approach and evaluation of patients with MDD. Understanding the inflammatory basis of MDD can aid in the identification of specific biomarkers of inflammation such as IL-6 and CRP to provide a more personalized treatment approach and informed therapeutic strategy in patients with Treatment Resistant Depression (TRD) (Jha and Trivedi, 2018). Previous studies have identified that higher levels of CRP, a nonspecific marker of inflammation, are associated with a greater likelihood of hospitalization due to MDD, increased severity of MDD, and a greater likelihood of completed suicide (Tonelli et al., 2008). A study conducted by Uher et al. (2014) found that patients with CRP < 1mgI-1 showed a greater symptomatic improvement in depression with escitalopram than with Nortriptyline, while those with CRP >1 showed greater improvement with nortriptyline rather than with escitalopram. Drevet’s demonstrated that those who received Anti-IL6 antibody Sirukumab as an adjunct treatment for TRD and had baseline CRP levels of >6mgI-1 showed significant improvements in anhedonia rating scales compared to controls at 12 weeks. Those with baseline CRP values of greater than 8mgI-1 showed a significant decrease in Hamilton Depression Rating Scale scores with Sirukumab as an adjunct treatment (Drevets et al., 2022). These studies among others exemplify a need for more personalized treatment options for those with MDD that is not responsive to conventional methods. In the future, inflammatory levels may become essential in the way we assess and manage patients with MDD (Tonelli et al., 2008; Lindqvist et al., 2009; Steiner et al., 2011; Pandey et al., 2012; Brundin et al., 2017).

## Immune pathways

Before studying the correlation between inflammation and psychiatry, we must first review some of the key inflammatory/immune players at the cellular level. Our immune system can be divided into innate and adaptive immune systems. The main cells comprising the innate immune system are granulocytes, mast cells, monocytes, dendritic cells, macrophages, and natural killer cells (Leo et al., 2011). The adaptive immune system is composed of cluster differentiation (CD) 4 T cells, CD8 T cells, B cells, and plasma cells (Leo et al., 2011). The key immune cells in our central nervous system are microglia cells (Nemeroff and Duman, 2015).

Some critical proteins that aid in signaling an immune response belong to the family of cytokines and include interleukins, interferons, and tumor necrosis factors. C-reactive protein (CRP), interleukin 1 (IL-1), interleukin 2 (IL-2) interleukin 6 (IL-6), tumor necrosis factor-alpha (TNF-α), and interferon-alpha (IFN-α), interferon-gamma (IFN-γ) are cytokines that have been correlated in pathways leading to mental illness and will be briefly discussed in this paper (Levine et al., 1999; Musselman et al., 2001; Lindqvist et al., 2009; Dowlati et al., 2010; Wium-Andersen et al., 2013; Currier, 2015).

Several studies have indicated that inflammation can precipitate depressive symptoms in patients treated with IFN-α or IL-2 (Renault et al., 1987; Buter et al., 1993; Janssen et al., 1994; Capuron et al., 2004; Dieperink et al., 2004). Approximately 30–45% of patients treated with interferon developed depressive symptoms during treatment and even after treatment completion. The purpose of this article is to demonstrate how cytokines including interferon affect the biochemical pathway of serotonin production (Meyers et al., 1991; Miyaoka et al., 1999).

Clinical and preclinical studies have shown that these same proinflammatory cytokines, namely IL-1B, IL-6, and TNF-α contribute to depressive symptoms (Raison et al., 2006; Franklin et al., 2018). Furthermore, other key molecules associated with the development of MDD are damage-associated molecular patterns (DAMPs), pathogen-associated molecular patterns (PAMPs), and toll-like receptors (TLRs), DAMPs are endogenous molecular patterns that are released secondary to physical and psychological stress and can stimulate inflammatory pathways even in the absence of pathogens (Franklin et al., 2018). PAMPs are molecules that are derived from pathogen invasion. TLRs are a class of protein recognition receptors (PRRs) that recognize PAMPs from diverse microorganisms and perform a key function in the innate immune system (Franklin et al., 2018). PAMPs are unique to various classes of microorganisms including viruses, bacteria, fungi, and protozoa but some PAMPs are common across various classes of pathogens, these are recognized by various PRRs (Franklin et al., 2018). These PRRs engage a series of downstream signaling pathways that trigger the activation of the innate immune response and the production of inflammatory cytokines (Franklin et al., 2018; Li and Wu, 2021).

Several studies have examined the development of MDD in the context of medical conditions with a high inflammatory burden. Some of these medical conditions will be discussed below. Some studies have investigated the development of MDD in the context of sterile inflammation, namely in otherwise medically healthy patients.

## Inflammatory markers and pro-inflammatory cytokines

Most evidence associating depressive symptoms with an inflammatory process includes three main concepts. (1) Systemic diseases with inflammatory processes increase the risk of depression. (2) Elevated pro-inflammatory markers are found in depressed patients. (3) Pro-inflammatory agents often can induce psychiatric side effects (Köhler et al., 2016).

(1) Systemic diseases with inflammatory pathophysiology have been associated with an increased risk of depression. These include rheumatoid arthritis, irritable bowel syndrome (IBS), coronary vascular disease (CVD), diabetes mellitus (DM), hepatitis c, and sepsis. We have reviewed some of these associations in this paper (Köhler et al., 2016).
(2) The associated relationship between proinflammatory markers and depression seems bidirectional since increased markers have been associated with the subsequent development of depression. Similarly, pro-inflammatory markers are found elevated in depressed patients independent of comorbid somatic disease. Specifically, IL-6, CRP, TNF-α, and IL-1 receptor antagonist (IL-1α). Elevated IL-6 levels during childhood have been associated with increased depression in young adults. Setiawan et al. found increased levels of neuroinflammation in individuals suffering from an active depressive episode (Setiawan et al., 2015; Köhler et al., 2016).
(3) The development of depressive disease has been found in patients treated with IFN-α in about 80% of patients. Sarkar et al. found antidepressant treatment before IFN-α treatment lowered mean depression scores and had a lower incidence of MDD (Sarkar and Schaefer, 2014; Köhler et al., 2016).

Given the above, anti-inflammatory interventions may represent opportunities for a more personalized treatment regimen in certain subgroups of depressed patients as discussed below (Köhler et al., 2016).

### Medical conditions in association with depression

Depression and inflammation can be seen in the context of several medical conditions with elevated inflammatory markers namely type 2 DM, CVD, hepatitis c, rheumatoid arthritis, and IBS. It can also be seen in the absence of medical conditions as sterile inflammation (Table 1).

**Table 1:** Medical conditions associated with MDD.

Study	Condition	Summary
Laake et al. (2014)	MDD in association with type 2 DM	More overweight, younger, higher levels of CRP, IL-1rα, and white-cell counts in patients with depression.
Hayashino et al. (2014)	MDD in association with high BMI	Depression and increased CRP were statistically significantly correlated
Lichtman et al. (2014)	MDD in association with type 2 DM	Incidence of dementia is increased in type 2 diabetics with depression
Duivis et al. (2013)	MDD in association with stable coronary heart disease	Depressive symptoms independently predicted a higher occurring WBC count in these patients. Which is linked to an increased risk of atherosclerosis and cardiac death
Goldstein et al. (2015)	MDD in association with CVD	The higher risk of MDD in CVD may be caused by chronic low-grade inflammation related to increased levels of IL-6 and CRP
Kudlow et al. (2013)	MDD in association with metabolic disease	Both MDD and metabolic problems have been independently linked to increased IL-6.
Cheng et al. (2021)	MDD in association with Hepatitis C	Kynurenine pathway has been linked to IFN-α-induced depression in patients with hepatitis C.
Kraus et al. (2000)	MDD in association with Hepatitis C	57.7% of the interferon treatment group was reported to develop clinically relevant mood disturbances during the therapy, compared to 22.5% before therapy.
Nerurkar et al. (2019)	MDD in association with Rheumatoid Arthritis (RA)	The increased prevalence of depression in RA patients may be partially explained by the involvement of several immunological changes linked to RA such as upregulation of IL-6 and TNF-α are in depressive illness.

One area that has received attention over recent years is the gut microbiome and the gut-brain axis. Immune system dysfunction, which is present in both IBS and depression, can be attributed to alterations in the permeability of the gastrointestinal tract and microbial characteristics. The complex multifactorial systems underlying IBS and depression are mediated by altered cytokines, immunological function, and changes in the HPA axis in response to stress. TNF-α and IL-8 levels that are abnormal are linked to depression. At the same time, depression alone changes IL-1 and IL-10 levels, generating an imbalance between pro- and anti-inflammatory markers, which results in a deviation that can cause IBS symptoms to appear and/or persist (Mudyanadzo et al., 2018). When an immunological response is triggered, psychological stress can make the intestinal lining more permeable, which makes it possible for bacterial liposaccharides (endotoxins) to enter the bloodstream (Peirce and Alviña, 2019). This peripheral inflammation can then move to the central nervous system (CNS) in a variety of ways, impairing neurotransmitters and encouraging neurotoxins, which have an impact on mental health (Peirce and Alviña, 2019). Peirce et al. summarized the mechanisms in the gut microbiome-brain axis that may affect CNS function. The intestinal barrier is strengthened by short-chain fatty acids (SCFAs), tryptophan catabolites (Trycats), and microbial-associated molecular patterns (MAMPs), which stop endotoxins from penetrating the intestinal epithelium, thus decreasing inflammation. Also, the vagus nerve communicates with the gut epithelium, including enteroendocrine cells providing an antidepressant effect. Probiotics, prebiotics, and fecal microbiota transplantation (FMT) have been shown to benefit both the gut microbiome and mental health.

Several medical conditions are associated with higher levels of inflammation. However, some examples of inflammation can be found in the following studies:

Stewart et al. (2009) studied the longitudinal association between depressive symptoms and both IL-6 and CRP among 263 healthy older patients enrolled in the Pittsburgh Healthy Heart Project, a 6-year prospective cohort study. Participants were evaluated for depressive symptoms using the Beck Depression Inventory-II (BDI-II) and had blood work quantifying IL-6 and CRP at baseline and during follow-up visits. Pathology analyses demonstrated that baseline BDI-II was a predictor for a 6-year change in IL-6.

Evidence indicates that MDD patients have elevated levels of circulating blood cytokines (Howren et al., 2009; Dowlati et al., 2010; Valkanova et al., 2013; Haapakoski et al., 2015). Several meta-analyses have found positive correlations between blood levels of both CRP and IL6 with depression (Valkanova et al., 2013; Haapakoski et al., 2015).

Other studies have reported strong associations between depression symptoms and circulating levels of TNF and IL-1β (Howren et al., 2009; Dowlati et al., 2010).

Acute and chronic stress promotes elevations of pro-inflammatory cytokines, including IL-1β, TNF, and IL-6 (You et al., 2011; Wohleb et al., 2012; Hodes et al., 2014; Şahin et al., 2015; Cheng et al., 2016).

Stress-induced sterile inflammation promotes the development of depressive-like behaviors in preclinical stress models of depression. For instance, Iwata et al. demonstrated that the development of stress-induced depressive-like behaviors requires NFkB activation and subsequent cytokine production (Koo and Duman, 2008, 2009; Koo et al., 2010; Iwata et al., 2013).

When reviewing the biochemical pathway of serotonin production we can identify specific steps on the pathway that are affected by inflammation. The information presented below will review the crucial steps in which inflammation can alter the production of serotonin suggesting a direct effect of inflammatory molecules on serotonin production. More clinical research is needed to elucidate altered levels of serotonin production in humans.

### Kynurenine pathway

Tryptophan (TRP) is an amino acid that is required for the production of serotonin. It can be metabolized via the serotonin pathway or the kynurenin (KYN) pathway. TRP is metabolized through the KYN pathway rather than the 5-HT pathway more than 90% of the time (Dantzer et al., 2008). The first step in the KYN pathway requires the conversion of TRP to KYN by two key enzymes, tryptophan 2,3-dioxygenase (TDO) and indoleamine 2,3 dioxygenase (IDO). Interferon-gamma IFN-γ is a cytokine that regulates cellular processes through transcription and translation (Inserra et al., 2019). IFN-γ binds the IFN-γ receptor, which then induces IDO expression via JAK/STAT signaling (Mellor and Munn, 2004; Okamoto et al., 2007). As a result, by inhibiting IFN-STAT1 activation, TRP may suppress IDO production at the transcriptional level. Additionally, the IFN-γ receptor also secondarily activates transcription factors which then leads to the induction of inflammatory genes (Inserra et al., 2019). During inflammatory states, TDO is suppressed while IDO is activated (Maes et al., 2007). IFN-γ is found to be elevated in MDD patients (Inserra et al., 2019). This could play a major role in the induction/upregulation of IDO, subsequent TRP depletion, decreases in serotonin levels, and the increase in KYN pathway activity (Inserra et al., 2019).

**Figure 1:**
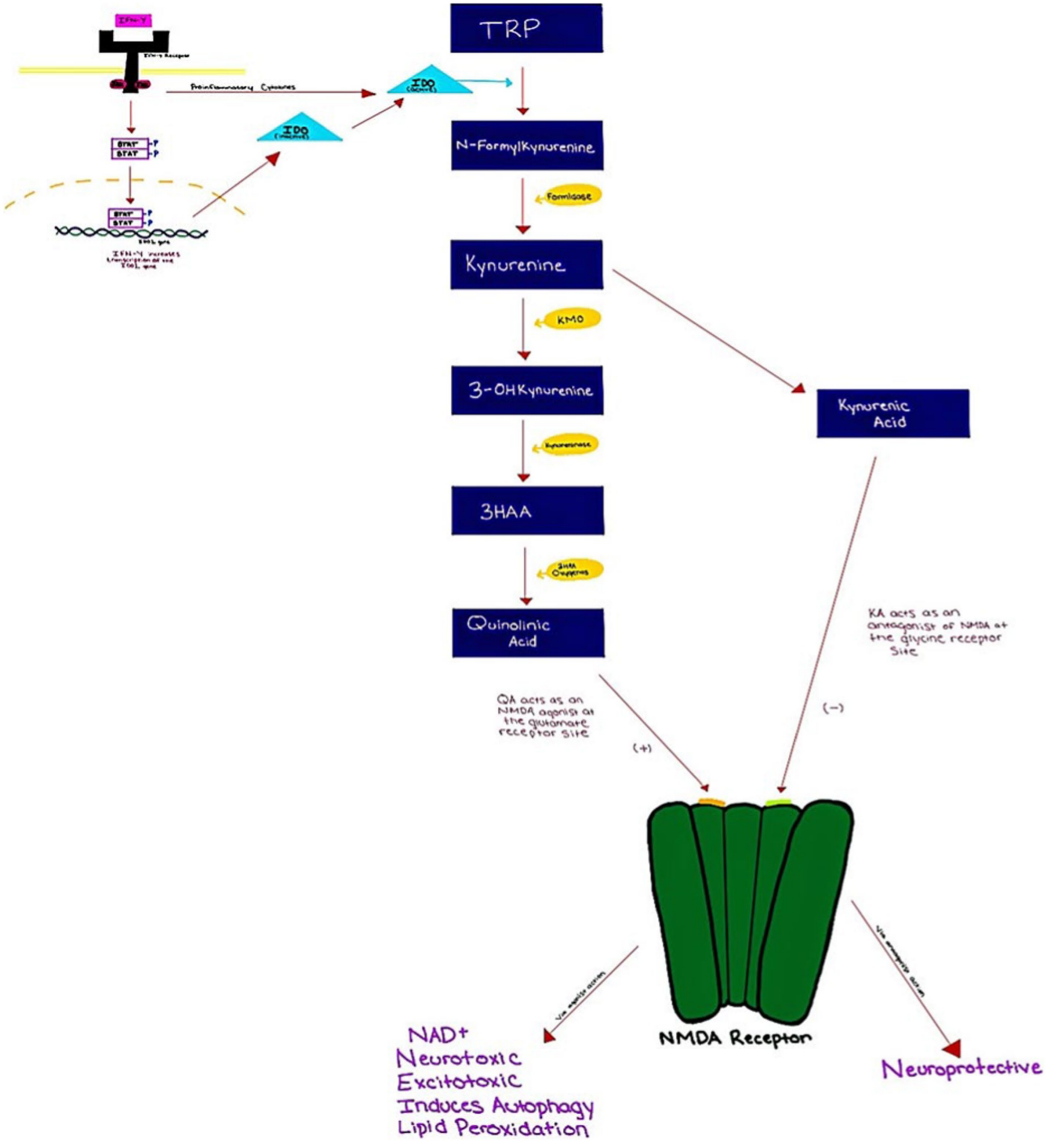
Kynurenine pathway from IFN-gamma activation to effects on the NMDA receptor.

KYN is converted to 3-hydroxykynurenine (3-HK) via kynurenine 3-monooxygenase (KMO) which is then metabolized into 3-hydroxyanthranilic acid (3-HAA) and quinolinic acid (QUIN) in microglia (Agudelo et al., 2014). Quinolinic acid is an agonist of the glutamate *N*-methyl-d-aspartate (NMDA) receptor and it has been associated with neurotoxicity, excitotoxicity, and preoxidative action (Tavares et al., 2002; Meier et al., 2016) (Figure 1). Past studies have demonstrated abnormal concentrations of kynurenic acid (KYNA) and QUIN, in patients with MDD (Réus et al., 2015; Filho et al., 2016; Więdłocha et al., 2018). These studies indicate increased levels of KYN byproducts during inflammation as opposed to serotonin. Hunt et al. (2020) in their systemic review and meta-analysis sought to examine the relation of KYN pathway metabolites such as TRP, KYN, KYNA, QUIN, and KYN/TRP ratio, in the context of pro-inflammatory activation and immune responses. This was conducted by analyzing 15 studies. Results indicated that there was an association between levels of KYN metabolites and pro-inflammatory activation, IFN-α treatment specifically showed an association between reduction of TRP levels and increase in KYN levels, and subsequently the KYN/TRP ratio. Increases in depression symptoms were also seen similar to increases in KYN metabolites.

Regarding the KYN pathway, possible mechanisms between metabolites and depressive symptoms are based on either the TRP depletion hypothesis or the toxic effects of KYN metabolites produced by KMO in the CNS. In the TPR depletion hypothesis, competition between the KYN pathway and the serotonin pathway for TRP uptake can lower TRP bioavailability. This also aligns with the serotonin hypothesis of depression. On the other hand, toxic KYN metabolites (QUIN in particular) mainly produced in microglia by KMO can be excitotoxic, altering glutaminergic activity, lipid peroxidation, and activating reactive oxygen species (ROS) in the CNS. It is important to note that the findings of this meta-analysis are mainly extended from medically ill patients who were treated with IFN-α and other immune-activating agents; how generalizable the associations drawn from them should be further investigated (Hunt et al., 2020).

### Toll-like receptors

Toll-like receptors (TLRs) also play a key role in the link between inflammatory pathways and the pathogenesis of depressive disorders. The TLR family has ten recognized receptors in humans: TLR1-TLR10 (Onofrio et al., 2020). These TLRs are essential for the detection of PAMPs on bacteria, viruses, and other parasites, cytokine modulation, and the induction of innate immunity (Hedayat et al., 2011; Birra et al., 2020; Debnath et al., 2020; Onofrio et al., 2020). TLRs are located on several immune cells, namely dendritic cells, macrophages, and natural killer cells (Khanmohammadi and Rezaei, 2021). TLRs 7/8 identify RNA viruses such as COVID-19 (Onofrio et al., 2020; Khanmohammadi and Rezaei, 2021). Activation of TLR 7/8 is followed by the release of IL-1, IL-6, TNF-α, and IFN-γ (Duivis et al., 2013). Several studies have investigated the association between COVID and depressive disorders. As research is underway looking at the correlation of pathogens causing depressive symptoms, more information and knowledge will be revealed about the intricate pathways connecting inflammation and mental illnesses. Hung et al. (2014) found TLR mRNA levels to be different in MDD patients compared to healthy controls. Also, TLR4 was discovered to be an independent risk factor for MDD severity. Hou et al. (2018) found that these TLR mRNA levels, which were higher in MDD patients, dropped after four weeks of treatment with SSRIs or SNRIs, implying that antidepressants have a TLR-mediated anti-inflammatory effect. They also showed that IL-6 mRNA expression in MDD patients’ was considerably reduced with an SSRI, but not an SNRI. These findings show that SSRIs and SNRIs influence TLR and cytokine expression differently and that various TLRs may be involved in MDD. Hajebrahimi et al. (2014) found the expression of adaptor proteins that mediate TLR signaling to be higher in 38 depressed medical students in comparison to healthy controls. When compared to healthy controls, depressed patients and postmortem brains had greater amounts of TNF, IL-6, TNF, and IL-1β. The rise in these cytokines could be due to TLR (1, 2, 4, 5, 6, and 10) activation on the cell surface (Kim et al., 2007; Yoshimura et al., 2009; Dowlati et al., 2010; Dahl et al., 2014; Köhler et al., 2018; Figueroa-Hall et al., 2020). TLRs and IL-1 receptors share homology. IL-1 cytokines modulate mu-opioid neurotransmission, this opioid neurotransmitter pathway has been demonstrated to be dysregulated in MDD (Prossin et al., 2016).

PAMPs linked to systemic infection have the potential to cause or worsen psychiatric disorders as evidenced by experimental studies by Irwin et al. (2019) who showed that low-dose lipopolysaccharide (LPS) can cause temporary depressed symptoms. Also, various studies have shown social withdrawal, reduced appetite, decreased motor activity, and altered cognition and sleep in mice administered LPS (a PAMP for TLR4) (Yirmiya, 1996; Yirmiya, 2000; Dantzer, 2001; Konsman et al., 2002; Figueroa-Hall et al., 2020). Lucas and Maes (2013) propose that a vicious loop between the TLR4 complex and the formation of ROS and reactive nitrogen species (RNS), which stimulates the TLR4 complex, might explain why chronic inflammation persists. They show many environmental variables can activate TLR4 pathways, resulting in inflammatory and oxidative, and nitrosative responses; they do so primarily through the actions of ROS/RNS-induced DAMPs on the TLR4 complex.

Several medications have been considered as potential anti-inflammatory agents that could augment antidepressant agents. Statins, corticosteroids, minocycline, modafinil, celecoxib, and infliximab have all been considered and investigated. Kohler et al. described several potential augmenting agents that could be used in managing depression (Köhler et al., 2014, 2016). The findings from studies supporting the use of anti-inflammatory agents for MDD treatment augmentation are summarized in the Table 2.

**Table 2:** Studies supporting the use of anti-inflammatory agents for MDD treatment augmentation.

Study	Type	Augmentin agent	Comorbid condition	Major finding
Müller et al. (2006)	Double-blind randomized placebo-control trial	Celecoxib		Improved antidepressant treatment effects
Akhondzadeh et al. (2009)	Double-blind placebo-control trial	Celecoxib		Improved antidepressant treatment effects
Abbasi et al. (2012)	Double-blind randomized placebo-control trial	Celecoxib		Improved antidepressant treatment effects. Higher levels of IL-6 predicted better antidepressant response to celecoxib add-on
Majd et al. (2015)	Double-blind randomized placebo-control trial	Celecoxib		Improved antidepressant treatment effects
Iyengar et al. (2013)	Double-blind randomized placebo-controlled clinical trial	ibuprofen vs. naproxen vs. celecoxib vs. placebo	Osteoarthritis	Ibuprofen, celecoxib or naproxen may have better antidepressant effects compared to placebo
Ghanizadeh and Hedayati (2013)	Double-blind randomized placebo-controlled clinical trial	fluoxetine with lovastatin vs. fluoxetine with placebo		Lovastatin augmentation was more effective
Gougol et al. (2015)	Double-blind placebo-controlled trial	simvastatin augmentation to fluoxetine		In patients with moderate to severe MDD simvastatin improved depressive symptoms
Miyaoka et al. (2012)	Open label study	Minocycline and fluvoxamine, paroxetine, or sertraline		Patients with MDD with psychotic features found significant improvement in depression
Tyring et al. (2006)	Double-blind placebo-controlled	Placebo vs. etanercept	Psoriasis	Etenercept effective for depression symptoms
Menter et al. (2010)	Randomized clinical trial	Placebo vs. adalimumab		Adalimumab effective for depression symptoms
Langley et al. (2010)	Double-blind randomized placebo-controlled	Placebo vs. ustekinumab		Ustekinumab effective for depression symptoms
Raison et al. (2012)	Randomized controlled trial	Placebo vs. infliximab		Infliximab effective for depression symptoms if CRP >5 mg/L
Abolfazli et al. (2011)	Double-blind randomized parallel group clinical trial	Modafinil + fluoxetine vs. placebo + fluoxetine		Modafinil found to be an effective augmentin agent
Goss et al. (2013)	Systematic review and meta-analysis	Modafinil or armodafinil		Modafinil found to be an effective augmentin agent

Since SSRI’s have been used for years to treat depression one can naturally wonder whether there is also a potential effect of SSRI’s on inflammation. Recent studies have shown that certain SSRI’s can have an effect on murine microglia response to inflammation (Hwang et al., 2008; Hashioka et al., 2009; Horikawa et al., 2010; Tynan et al., 2012). Further research is required and eagerly anticipated when exploring this topic.

### Bottom line

A considerable fraction of the global population suffers from a depressive disorder. It is a very complicated illness with a wide range of manifestations and many co-occurring medical and mental conditions. There has been a rigorous effort to better manage symptoms of MDD. We now know more about the underlying mechanisms of depression other than the monoaminergic hypothesis. Chronic low-grade inflammation has been associated with depression via many mechanisms as we discussed above. The pathways are more intricate than previously thought as our brain is undoubtedly incredibly complex. There are a multitude of studies describing the role that inflammation plays in MDD and this could explain why two-thirds of patients continue to struggle with depressive symptoms despite having tried multiple SSRI’s and SNRI’s. Given this body of evidence, it would be remiss to disregard this association. Current studies are examining immunotherapy treatments for MDD ultimately aiming to better manage patients with MDD (Evrensel et al., 2021; Drevets et al., 2022). As future studies continue to explore MDD in the context of inflammation new paths can be forged in the treatment of this disorder giving hope to patients with treatment-resistant MDD (Evrensel et al., 2021; Drevets et al., 2022).

## Author contributions

IP: Conceptualization, Investigation, Resources, Supervision, Writing – original draft, Writing – review & editing. MS: Conceptualization, Investigation, Resources, Writing – original draft, Writing – review & editing. AP: Investigation, Writing – original draft.
